# Generalizability of sodium-glucose co-transporter-2 inhibitors cardiovascular outcome trials to the type 2 diabetes population: a systematic review and meta-analysis

**DOI:** 10.1186/s12933-020-01067-8

**Published:** 2020-06-13

**Authors:** Marco Castellana, Filippo Procino, Rodolfo Sardone, Pierpaolo Trimboli, Gianluigi Giannelli

**Affiliations:** 1 Population Health Unit, National Institute of Gastroenterology “Saverio de Bellis”, Research Hospital, Castellana Grotte, Bari, Italy; 2 Clinic for Nuclear Medicine and Competence Center for Thyroid Diseases, Imaging Institute of Southern Switzerland, Ente Ospedaliero Cantonale, Bellinzona, Switzerland; 3 Scienze Biomediche, Università della Svizzera Italiana (USI), Lugano, Switzerland; 4 National Institute of Gastroenterology “Saverio de Bellis”, Research Hospital, Castellana Grotte, Bari, Italy

**Keywords:** Cardiovascular outcome trials, Generalizability, Type 2 diabetes mellitus, Lifestyle, Meta-analysis

## Abstract

**Background:**

Cardiovascular outcome trials of sodium-glucose co-transporter-2 inhibitors (SGLT2i CVOTs) found the agents to be associated with clinical benefits in terms of cardiovascular and renal outcomes. We performed a meta-analysis to assess and compare the overall prevalence of eligibility for the enrollment criteria of CANVAS, DECLARE-TIMI 58, EMPA-REG OUTCOME, and VERTIS-CV among unselected patients with type 2 diabetes.

**Methods:**

This meta-analysis was registered in PROSPERO (CRD42020172032). PubMed, CENTRAL, Scopus and Web of Science were researched in March 2020. Studies evaluating the prevalence of eligibility for each SGLT2i CVOT were selected. Endpoints were estimated using a random-effects model.

**Results:**

Five studies, evaluating 1,703,519 patients with type 2 diabetes, were included. Overall, the prevalence of eligible patients according to the enrollment criteria of CANVAS, DECLARE-TIMI 58, EMPA-REG OUTCOME, and VERTIS-CV was 36.4%, 49.5%, 17.0% and 19.0%, respectively. In head-to-head comparisons, DECLARE-TIMI 58 was associated with the highest odds of eligibility (1.74 versus CANVAS, 5.15 versus EMPA-REG OUTCOME and 4.81 versus VERTIS-CV), followed by CANVAS and EMPA-REG OUTCOME/VERTIS-CV. A high heterogeneity was found for all the outcomes.

**Conclusions:**

The present review showed that a considerable number of patients counseled in clinical practice could have been eligible for SGLT2i CVOTs. Particularly, dapagliflozin was shown to be the SGLT2i with the largest generalizability of findings from its CVOT according to the odds ratio of eligibility for the enrollment criteria among unselected patients with type 2 diabetes. Further country- or region-specific studies are needed to confirm the applicability of our results.

## Background

Diabetes is a complex chronic disease characterized by high prevalence, morbidity, and excess mortality [1]. To reduce the burden of this disease as well as its economic impact on people with diabetes, their families and the health care system, current guidelines strongly recommend that all the components of the metabolic syndrome should be targeted. Intensive lifestyle measures, pharmacological therapy or other interventions (e.g. bariatric surgery) should be considered aiming at blood glucose concentrations, blood pressure and lipids levels in line with the best available standard of care as well as weight loss in subjects with overweight and obesity [2–7]. Concerning drugs for the management of type 2 diabetes, the findings of several cardiovascular outcome trials (CVOTs) have recently been published. While CVOTs of dipeptidyl peptidase-4 inhibitors (DPP-4i) reported that these agents neither increased nor decreased cardiovascular events, findings from studies of sodium-glucose co-transporter-2 inhibitors (SGLT2i) and glucagon-like peptide-1 receptor agonists (GLP-1RA) demonstrated benefits on cardiovascular and renal outcomes, with some differences [8–10]. In the light of these results, international and national societies have updated their guidelines for type 2 diabetes pharmacological therapy and recommended the preferential use of SGLT2i and/or GLP-1RA in patients with indicators of a high-risk or history of cardiovascular disease, heart failure or chronic kidney disease [11, 12].

In a patient for whom the use of a SGLT2i is indicated, the evidence-based choice of the specific drug can be an issue. The efficacy and safety on cardiovascular outcomes of canagliflozin, dapagliflozin, empagliflozin and ertugliflozin were assessed in CANVAS, DECLARE-TIMI 58, EMPA-REG OUTCOME, and VERTIS-CV, respectively [13–16]. These large double-blind, multinational, placebo-controlled trials were performed to satisfy regulatory requirements of cardiovascular safety (non-inferiority) and to test efficacy (superiority) under similar glucose levels between treatment and placebo groups [13–16]. A number of patients with type 2 diabetes ranging from 7020 in EMPA-REG OUTCOME to 17,160 in DECLARE-TIMI 58 were included; they were followed-up for a median period ranging from 2.4 years in CANVAS to 4.2 years in DECLARE-TIMI 58 [15, 16]. To date, a report release only is available for VERTIS-CV. While the primary endpoint of non-inferiority was met, key secondary endpoints of superiority were not; detailed results are scheduled to be presented in June 2020 [13, 17]. Concerning the other SGLT2i, similar overall trends in the relative risk reductions for hospitalization for heart failure, renal outcomes and cardiovascular events have been found among CANVAS, DECLARE-TIMI 58 and EMPA-REG OUTCOME [14–16]. A class effect has been suggested, accordingly, and the use of canagliflozin, dapagliflozin or empagliflozin has been recommended with a similar strength of evidence [11, 12, 18, 19].

It is worth noting that these SGLT2i CVOTs differed in terms of results and/or design [20]. The EMPA-REG OUTCOME was the only SGLT2i CVOT to show a reduction in death from cardiovascular causes and from any cause (hazard ratio [HR] = 0.62; 95% confidence interval [95% CI] 0.49 to 0.77 and HR = 0.68; 95% CI 0.57 to 0.82, respectively) [16]. Also, the primary outcome on the 3-point major cardiovascular events was met only in CANVAS (HR = 0.86; 95%CI 0.75 to 0.97) and EMPA-REG OUTCOME (HR = 0.86; 95% CI 0.74 to 0.99) [14, 16]. As a relevant aspect, patients without a history of cardiovascular disease represented 34% and 59% in CANVAS and DECLARE-TIMI 58, respectively, while they accounted for less than 1% in the EMPA-REG OUTCOME [14–16]. Randomized controlled trials, including SGLT2i CVOTs, are generally developed to assess the efficacy and safety of a therapeutic agent under ideal conditions. Only those patients with the highest chance of showing an effect, if any, tend to be selected while up to 96% of subjects with the condition being treated may be excluded, introducing a possible selection bias [21, 22]. This raises the question as to whether the included patients are representative of the general population of patients with type 2 diabetes and so the results of these studies are applicable in clinical practice.

To address this issue, several papers attempted to compare the generalizability of SGLT2i CVOTs [23–27]. There, two specific outcomes were assessed: the prevalence of eligible patients according to each CVOT protocol and the differences in the characteristics between eligible patients from each analytic cohort and those included in each CVOT. The results of those studies have been heterogeneous, thus limiting their generalizability to the type 2 diabetes general population. Moreover, while some studies assessed general registries, others included only patients initiating SGLT2i in the real-world practice [23, 26]. Consequently, these studies were affected by a different selection bias, which in turn impacted on the prevalence of cardiovascular disease [23–27]. It is well known that absolute measures are of limited generalizability due to their dependence on the baseline values [28]. As a proof, significant discrepancies were observed in terms of the prevalence of eligible patients according to each CVOT protocol in those studies [23–27]. In order to overcome these limitations, relative measures assumed to be stable across populations with different characteristics should be used (e.g. odds ratio) [28]. Indeed, while the former only allows the results within a study to be evaluated, the latter allows the findings to be projected to populations other than those included in the analysis [28]. Accordingly, we conducted the present study to gain information about the eligibility to the enrollment criteria for each SGLT2i CVOT among unselected patients with type 2 diabetes. With this aim, we designed a systematic search to identify studies reporting data on the prevalence of eligibility. Additionally, we performed a meta-analysis of available data to: (1) determine the prevalence of eligible patients according to each CVOT protocol; and (2) compare the prevalence of eligibility among CVOTs with a head-to-head approach.

## Methods

The meta-analysis was registered in PROSPERO (CRD42020172032) and performed in accordance with the PRISMA statement (see Additional file 1) [29].

### Search strategy

A six-step research strategy was drawn up. Firstly, we searched for sentinel studies in PubMed, in the sense of those studies considered fundamental to our systematic review, as described in more detail below. Secondly, we identified keywords in PubMed. Thirdly, the following complete search strategy was used in PubMed: (applicability[All Fields] OR eligibility[All Fields] OR generalizability[All Fields]) AND cardiovascular[All Fields] AND outcome[All Fields] AND diabetes[All Fields]. Fourthly, CENTRAL, Scopus and Web of Science were researched using the same strategy. Fifthly, studies evaluating the eligibility for the enrollment criteria of SGLT2i CVOTs among patients with type 2 diabetes were selected. Studies including only patients with a history of cardiovascular disease, heart failure or chronic kidney disease were excluded, since findings from these studies would not have been generalizable to the type 2 diabetes population. Lastly, references of the included studies were searched to find additional papers. The last search was performed on March 3, 2020. No language restriction was adopted. Two investigators (MC, FP) independently searched for papers, screened titles and abstracts of the retrieved articles, reviewed the full-texts, and selected the articles for inclusion.

### Data extraction

The following information was extracted independently by the same investigators in a piloted form: (1) general information on the study (author, year of publication, country, study type, number of patients, prevalence of cardiovascular disease); (2) evaluated SGLT2i CVOTs, among CANVAS for canagliflozin, DECLARE-TIMI 58 for dapagliflozin, EMPA-REG OUTCOME for empagliflozin, and VERTIS-CV for ertugliflozin [13–16]; (3) criteria assessed to determine the eligibility of subjects included in each study to each SGLT2i CVOT protocol; (4) number of patients eligible for the enrollment criteria of each SGLT2i CVOT. The main paper and supplementary data were examined. Data were cross-checked, and any discrepancy was discussed.

### Study quality assessment

The risk of bias of included studies was assessed independently by two reviewers (MC, FP) using the National Heart, Lung, and Blood Institute Quality Assessment Tool. Fourteen criteria were assessed, using detailed instruction for rating. The following aspects were evaluated: study question; eligibility criteria; sample size calculation; exposure of interest; outcome of interest; blinding; loss to follow-up; statistical methods. Each domain was rated as absent, unclear or with a possible risk of bias, or as not applicable [30].

### Data analysis

The primary outcome was the difference in eligibility for the enrollment criteria of CANVAS, DECLARE-TIMI 58, EMPA-REG OUTCOME, and VERTIS-CV among unselected patients with type 2 diabetes. Firstly, a meta-analysis of proportions was carried out to obtain the pooled prevalence of eligibility for each SGLT2i CVOT. Secondly, a head-to-head comparison of the prevalence of eligibility for each SGLT2i CVOT was performed if at least three studies were available. By using this approach, only data estimated using the same methods and from the same analytic cohorts were included. Two SGLT2i CVOTs were included in each comparison. Endpoints were analyzed as dichotomous variables, and proportions and odds ratios were estimated. Heterogeneity between studies was assessed by using I^2^, 50% or higher being regarded as high. Publication bias was assessed with Egger’s test; the trim-and-fill method was used for estimating its effect. Sensitivity analyses by removing each study in turn were also performed. All analyses were two-sided and were carried out using RevMan5.3 (The Cochrane Collaboration) and Prometa3.0 (Internovi) with a random-effect model; significance was set at p < 0.05.

## Results

### Study characteristics

**Fig. 1:**
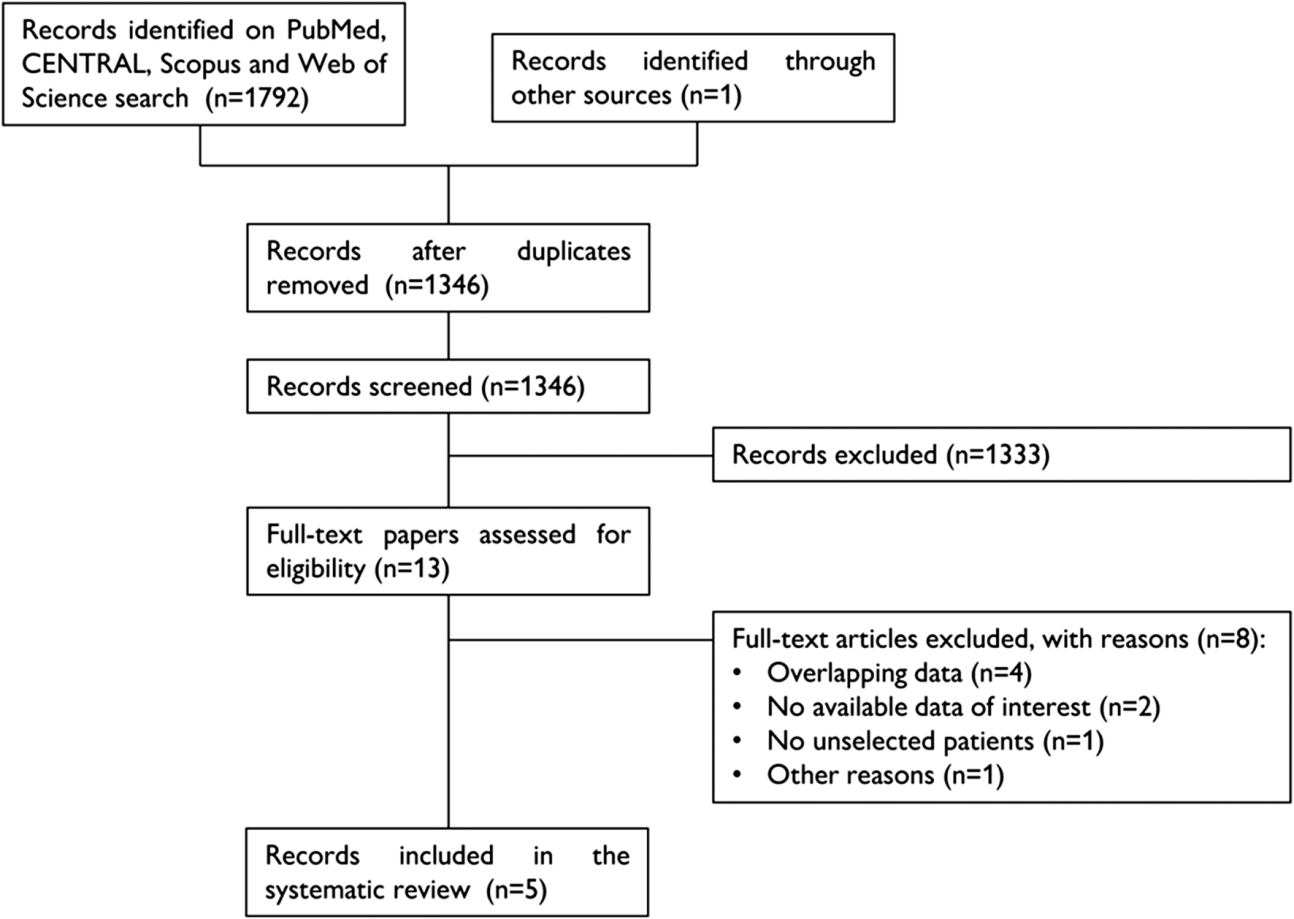
PRISMA flow diagram

In total, 1 792 papers were found, of which 210 on PubMed, 868 on CENTRAL, 391 on Scopus, and 323 on Web of Science. One additional paper was retrieved from a personal database [19]. After removing 447 duplicates, 1 346 articles were analyzed for title and abstract; 1 333 records were excluded (review, meta-analysis, study protocol, not on SGLT2i, not assessing eligibility for the enrollment criteria of SGLT2i CVOTs, commentary, poster). The remaining 13 papers were retrieved in full-text and five studies were finally included in the meta-analysis (Fig. 1) [19–23]. No additional study was retrieved from the references of included studies.

### Study quality assessment

The risk of bias of the included studies is shown in Additional file 2. Statement of the study question and description of the study population were adequate in all. The participation rate was below 50% in one study for CANVAS since only patients with no missing information about all major eligibility criteria could be included [25]. For the same reason, the number of patients assessed for the eligibility for each CVOT differed in the same study [25]. No sample size justification was reported in any study. As regards exposure measures, they were generally clearly defined, valid, reliable, and implemented consistently across all study participants. The only exception was the study by Birkeland et al. [23] in which data on body mass index (BMI) and tobacco use were not included, while laboratory data were included only in a sensitivity analysis on data from the Netherlands. As to outcome measures, eligibility for CVOTs was clearly reported as the endpoint in all the studies. Exposure of interest, timeframe, level of exposure of interest, repeated exposure assessment, blinding, and confounding variables bias were rated as not applicable, due to the design of the included studies.

### Qualitative analysis

The characteristics of the included articles are summarized in Table 1 [23–27]. The studies were published between 2018 and 2019 and had sample sizes ranging from 11 650 to 803 836 patients. One study was carried out in Italy, one in Spain, one in Taiwan, one in the United States of America, and one in Germany, the Netherlands, Norway and Sweden. Four studies assessed the eligibility for the enrollment criteria of all four included SGLT2i CVOTs, while one evaluated the CANVAS, DECLARE-TIMI 58 and EMPA-REG OUTCOME only [24]. Methods for the assessment of the eligibility for the enrollment criteria were similar, with the exception of the Birkeland et al. [23]study, as stated (see Additional files 3 and 4). Data were derived from diabetes-specific registries in three studies [24, 25, 27] and other healthcare databases in two studies [23, 26]. The prevalence of cardiovascular disease ranged from 23 to 64% [24, 27]. Other baseline characteristics of analytic cohorts were heterogeneously or not extensively reported [23–27]. Overall, 1 703 519 patients with type 2 diabetes were included.

**Table 1:** Characteristics of included studies and evaluated sodium-glucose co-transporter-2 inhibitors cardiovascular outcome trials

First Author, year	Country	Study design	Patients (n)	Cardiovascular disease (%)	CANVAS	DECLARE-TIMI 58	EMPA-REG OUTCOME	VERTIS-CV
Birkeland, 2018 [23]	Germany, the Netherlands, Norway, Sweden	RCS	803,836	34	○	○	○	○
Canivell, 2019 [24]	Spain	RCS	373,185	23	○	○	○	–
Nicolucci, 2019 [25]	Italy	RCS	342,205	–	○	○	○	○
Shao, 2019 [26]	Taiwan	RCS	11,650	27	○	○	○	○
Wittbrodt, 2019 [27]	the United States of America	RCS	172,643	64	○	○	○	○

*RCS* retrospective cohort study; – not reported

### Quantitative analysis

**Fig. 2:**
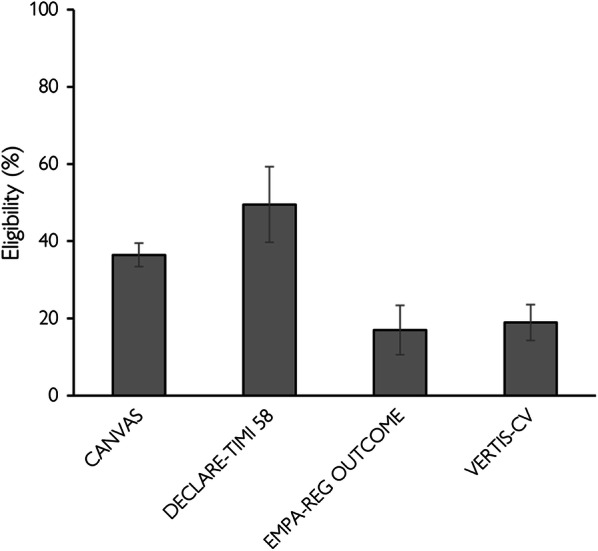
Prevalence of eligible patients according to the enrollment criteria of CANVAS, DECLARE-TIMI 58, EMPA-REG OUTCOME, and VERTIS-CV

The prevalence of eligible patients in the analytic cohorts according to the enrollment criteria of CANVAS, DECLARE-TIMI 58, EMPA-REG OUTCOME, and VERTIS-CV was 36.4%, 49.5%, 17.0% and 19.0%, respectively (Table 2, Fig. 2).

**Table 2:** Prevalence of eligible patients according to the enrollment criteria of CANVAS, DECLARE-TIMI 58, EMPA-REG OUTCOME, and VERTIS-CV

SGLT2i CVOT	Number of patients (number of studies)	Prevalence (95% CI)	I^2^ (%)
CANVAS	1,510,378 (5)	36.4 (33.3 to 39.4)	100
DECLARE-TIMI 58	1,619,008 (5)	49.5 (39.7 to 59.3)	100
EMPA-REG OUTCOME	1,703,519 (5)	17.0 (10.6 to 23.4)	100
VERTIS-CV	1,330,334 (4)	19.0 (14.4 to 23.7)	100

SGLT2i CVOT, sodium-glucose co-transporter-2 inhibitor cardiovascular outcome trial

**Fig. 3:**
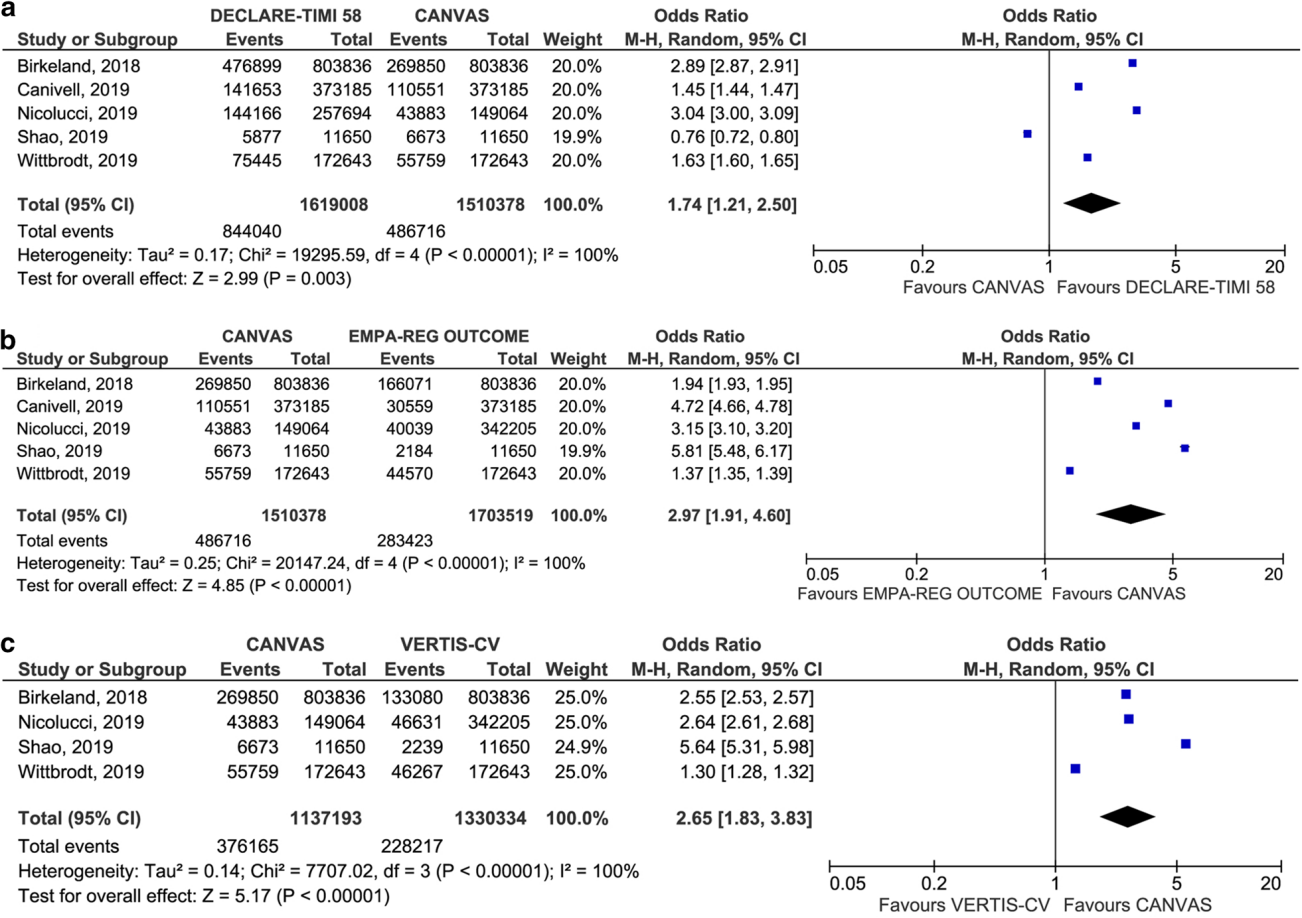
Forest plot of meta-analysis for the head-to-head comparison of eligibility for the enrollment criteria of CANVAS and DECLARE-TIMI 58 (**a**), CANVAS and EMPA-REG OUTCOME (**b**), and CANVAS and VERTIS-CV (**c**)

**Fig. 4:**
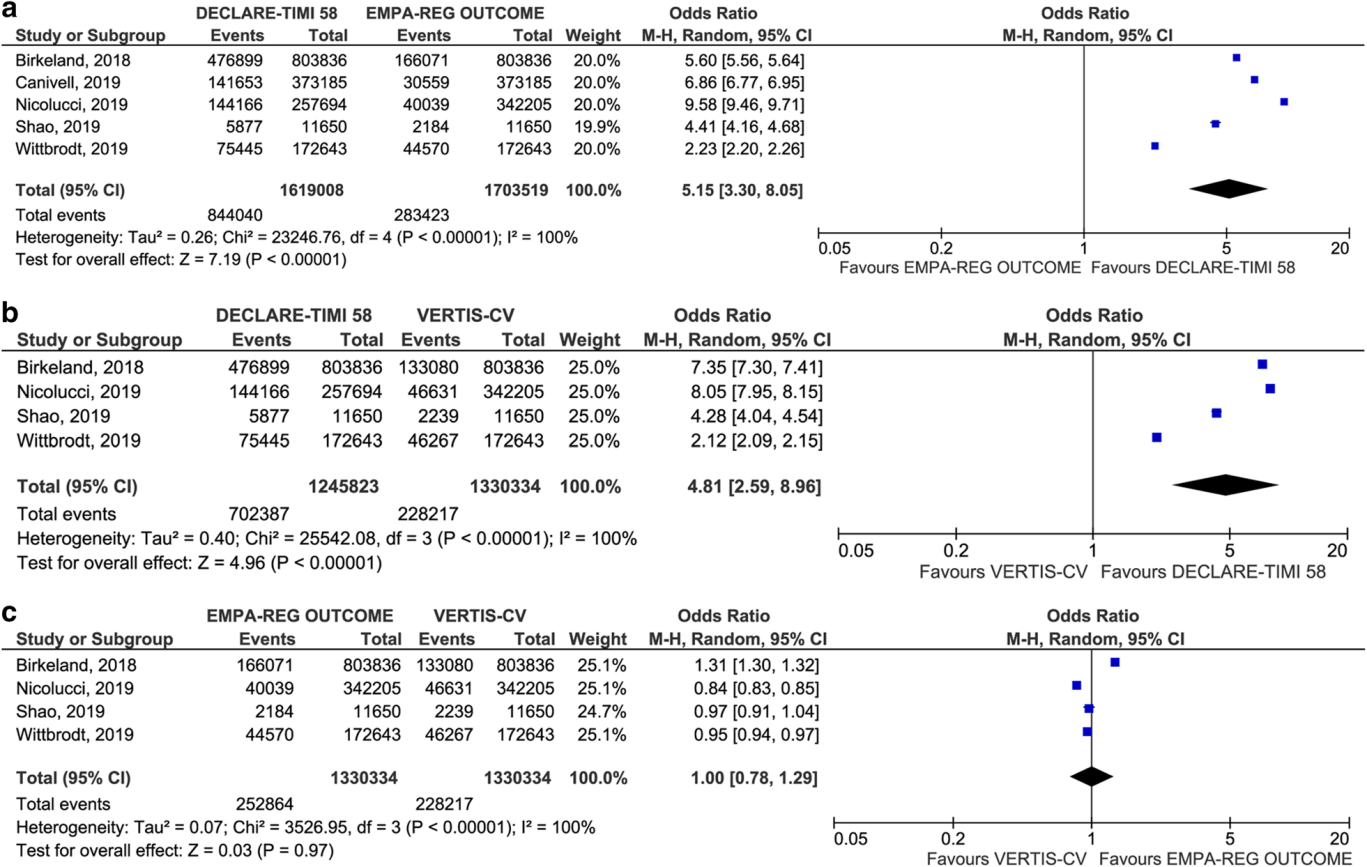
Forest plot of meta-analysis for the head-to-head comparison of eligibility for the enrollment criteria of DECLARE-TIMI 58 and EMPA-REG OUTCOME (**a**), DECLARE-TIMI 58 and VERTIS-CV (**b**), and EMPA-REG OUTCOME and VERTIS-CV (**c**)

Since the external validity of absolute measures is limited, we estimated the following relative parameters, which are assumed to be stable and potentially applicable across populations with different characteristics [28]. In head-to-head comparisons of the prevalence of eligibility for each SGLT2i CVOT, DECLARE-TIMI 58 was associated with the highest odds of eligibility, followed by CANVAS and EMPA-REG OUTCOME/VERTIS-CV. The odds for eligibility for the enrollment criteria of DECLARE-TIMI 58 were 1.74 (95% CI 1.21 to 2.50; p = 0.003) versus CANVAS, 5.15 (95% CI 3.30 to 8.05; p < 0.001) versus EMPA-REG OUTCOME and 4.81 (95% CI 2.59 to 8.96; p < 0.001) versus VERTIS-CV. No difference was found between EMPA-REG OUTCOME and VERTIS-CV (p = 0.97) (Figs. 3 and 4). A high heterogeneity was found for all the outcomes. There was no evidence of publication bias (see Additional file 5). Results were generally confirmed in the sensitivity analyses; the only exception was a non-statistically significant odds ratio in the head-to-head comparison of eligibility for the enrollment criteria of DECLARE-TIMI 58 versus CANVAS after removing one study [25] (see Additional file 6).

## Discussion

The aim of this meta-analysis was to identify the best available evidence of the generalizability of the findings of SGLT2i CVOTs to the general population of patients with type 2 diabetes. Particularly, we aimed to assess the prevalence of eligible patients according to the enrollment criteria of CANVAS, DECLARE-TIMI 58, EMPA-REG OUTCOME, and VERTIS-CV and whether any one trial was associated with a higher chance of eligibility among unselected patients with type 2 diabetes. To our knowledge, this is the first meta-analysis on the topic. We believe this to be a significant contribution to the current understanding, since studies evaluating populations from different countries could be interpreted together. An extensive database search was performed without time or language restrictions and inclusion criteria had been defined prior to the database search. Five studies were found, including a total of 1 703 519 patients with type 2 diabetes.

The effectiveness of SGLT2is in patients with type 2 diabetes in terms of glycemic control and extra-glycemic outcomes has been validated in several clinical trials [31–33], real-world studies [34, 35] and meta-analyses [9, 36–38]. Findings from CVOTs support their use to achieve relevant clinical benefits in terms of cardiovascular and renal outcomes. Of note, this property seems to be preserved even when SGLT2i are combined with GLP-1RA, as confirmed by a recent post hoc analysis of the EXSCEL trial [39]. We found that the prevalence of eligible patients in the analytic cohorts ranged from one in two to one in five according to the criteria of DECLARE-TIMI 58 and EMPA-REG OUTCOME, respectively. Therefore, enrollment criteria should be considered generally adequate from a clinical perspective and the findings of CVOTs potentially applicable to a relevant number of patients. These estimates were based on absolute measures (e.g. prevalence) which are characterized by a limited applicability to populations other than those evaluated [28]. Indeed, included studies were performed in a limited number of countries and their analytical cohorts represented only a part of the country-specific patients with type 2 diabetes, ranging from 0.6% in Wittbrodt et al. [27] to 9.7% in Birkeland et al. [23] (Additional file 7). Therefore, in order to assess differences between SGLT2i CVOTs, head-to-head comparisons based on relative measurements assumed to be stable across populations with different characteristics were performed. A higher odds ratio of eligibility for DECLARE-TIMI 58 was found, followed by CANVAS and EMPA-REG OUTCOME/VERTIS-CV. While the generalizability of findings of CVOTs varied among the assessed studies, dapagliflozin should be considered as the SGLT2i with the largest generalizability of findings from its CVOT.

What could be the reasons for these results? Firstly, this reflects the differences in the enrollment criteria of each CVOT. It is common knowledge that only patients with a history of cardiovascular disease could be enrolled in EMPA-REG OUTCOME and VERTIS-CV, while patients in primary prevention were eligible for CANVAS and DECLARE-TIMI 58, too. In the former group of studies, there were only some minor variations, including: (1) age, since EMPA-REG OUTCOME enrolled adults of any age, while only subjects aged ≥ 40 years were considered eligible for VERTIS-CV; (2) HbA1c, ranging from 7.0 to 10.0% versus 7.0 to 10.5%, respectively; and (3) BMI, since EMPA-REG OUTCOME excluded subjects with a BMI > 45 kg/m^2^, while VERTIS-CV included only those with a BMI ≥ 18 kg/m^2^. On the other hand, concerning the latter group of studies, while in CANVAS two or more risk factors (in addition to age) were needed for a patient without a history of a cardiovascular disease to be eligible, only one risk factor (in addition to age) among hypertension, dyslipidemia or tobacco use was enough for DECLARE-TIMI 58. Other differences included: (1) age, since subjects with established cardiovascular disease could have been eligible for CANVAS if ≥ 30 years versus ≥ 40 years for DECLARE-TIMI 58; (2) type of cardiovascular events, with no clear reference to patients with unstable angina in two trials [13, 15]; (3) type of risk factors. Furthermore, DECLARE-TIMI 58 was characterized by a wider range of HbA1c, from 6.5% to 12.0%, versus 7.0% to 10 or 10.5% in the other protocols. The cut-off for the renal function may also play a role. Despite a more restrictive cut-off reported in DECLARE-TIMI 58 (CrCl ≥ 60 versus eGFR ≥ 30 ml/min/1.73 m^2^), this did not result in a lower prevalence of eligibility. Whether this could be partially due to the different equations employed for the estimation of the renal function, as DECLARE-TIMI 58 adopted the Cockroft-Gault formula and CANVAS,EMPA-REG OUTCOME and VERTIS-CV the Modification of Diet in Renal Disease equation, is unclear (Additional file 3) [13–16, 40].

The present meta-analysis used relative measures assumed to be stable across populations, as stated, and this represents the basis for our results to be potentially projectable to populations other than those included in the analysis. Therefore, one may wonder what implications this could have on clinical practice. No data on the characteristics of patients who could have been selectively eligible for one specific SGLT2i CVOTs while excluded from the others have been reported, therefore we could not directly address the proportion of patients who should have been prescribed with a specific SGLT2i. Nevertheless, from a clinical perspective, the results of this meta-analysis introduce a high level of evidence on a novel aspect to be considered when making the evidence-based choice of the specific SGLT2i. We are used to considering the individual characteristics and comorbidities, individual preferences and class/drug-specific properties. However, how should we select the specific SGLT2i in a patient with uncontrolled type 2 diabetes, and predominant heart failure or chronic kidney disease? CANVAS, DECLARE-TIMI 58 and EMPA-REG OUTCOME all showed a benefit, then the use of canagliflozin, dapagliflozin or empagliflozin would be indicated with the same strength in this patient. Similar considerations can potentially be made for a patient with uncontrolled type 2 diabetes, without a history of cardiovascular disease, heart failure or chronic kidney disease [9, 12, 41, 42]. Based on our data, the use of dapagliflozin or canagliflozin could be preferred over empagliflozin or ertugliflozin in some circumstances.

Limitations of the present review should be considered. Firstly, a limited number of studies, evaluating patients from a limited number of countries, was found. Particularly, the databases representativeness of the overall population differed among the included studies, as stated. However, the total of patients evaluated amounted to 1 703 519 patients from Europe, Asia and North America. Also, our findings on the primary outcome were in line with the studies performed on data from DISCOVER, a large international, 3-year, prospective, observational study including 38 countries across six continents, and on data from National Health and Nutrition Examination Survey, the largest and longest-running survey of health and nutrition data for the U.S. population. These studies were excluded from the present meta-analysis due to overlap of countries and/or study period [43, 44]. Secondly, a high heterogeneity for all endpoints was found and this could be due to differences in the baseline characteristics of included subjects (see below) or in methods for the assessment of eligibility. Particularly, in Canivell et al. and in Wittbrodt et al. high rates of missing data for some parameters (e.g. HbA1c/eGFR and urine albumin/high-density lipoprotein cholesterol, respectively) were reported, possibly leading to an underestimation of trial eligibility [24, 27]. On the other hand, the assessment of exclusion criteria was not extensively reported, possibly leading to an overestimation of study findings [25]. Also, in Birkeland et al. [23] laboratory data were not included in the overall analysis. A sensitivity analysis was performed only on patients from the Netherlands and, when laboratory data were assessed, the prevalence of eligibility declined from 73 to 40% in DECLARE-TIMI 58, from 39 to 8% in CANVAS, from 13 to 4% in EMPA-REG OUTCOME, and from 7 to 2% in VERTIS-CV. Therefore, estimates on the prevalence of eligible patients according to each CVOT protocol in each study may possibly be biased. Nevertheless, the reliability of our findings on the head-to-head comparisons should not be influenced by the issues above, since based on relative measures and as confirmed by the sensitivity analyses. Thirdly, the baseline characteristics of analytic cohorts were heterogeneously or not extensively reported, including the prevalence of cardiovascular disease, as stated [25]. This limited our ability to perform additional analyses (for example a meta-regression to evaluate the relationship between the OR of eligibility and the prevalence of cardiovascular disease). Lastly, the enrollment criteria of SGLT2i CVOT do not always correspond to the approved indications and/or to the recommendations for the use of SGLT2i as a class or specific medication, which latter differ among countries and scientific societies [45, 46]. Caution should thus be used in generalizing these results to clinical practice, even if recent evidence from real-life studies and randomized controlled studies suggests that findings obtained in CVOT setting may be confirmed in clinical practice in subjects with different characteristics [47–51].

## Conclusions

Evidence from cardiovascular outcome trials supports the use of SGLT2i in patients with type 2 diabetes with a history of cardiovascular disease or risk factors. Evaluating the eligibility for the enrollment criteria of these trials of patients counselled in clinical practice is key to ensure the reproducibility of findings. The present meta-analysis found that from one in two to one in five patients could be eligible for the enrollment criteria. Particularly, DECLARE-TIMI 58 was associated with the highest odds ratio of eligibility for the enrollment criteria compared to CANVAS, EMPA-REG OUTCOME and VERTIS-CV. Therefore, dapagliflozin should be considered to be the SGLT2i with the largest generalizability of findings from its CVOT and this aspect taken into account when selecting the specific drug in a patient for whom the use of a SGLT2i is indicated. Further country- or region-specific studies are needed to confirm the applicability of our results.

## Supplementary information

**Additional file 1.** PRISMA Checklist.

**Additional file 2.** Risk of bias summary: review of authors’ judgements about each risk of bias item for each included study.

**Additional file 3.** Enrollment criteria for sodium-glucose co-transporter-2 inhibitors cardiovascular outcome trials.

**Additional file 4.** Enrollment criteria evaluated in each study.

**Additional file 5.** Publication bias.

**Additional file 6.** Sensitivity analysis.

**Additional file 7.** Estimated representativeness of the analytical cohorts of included studies.

## Data Availability

The datasets used and/or analysed during the current study are available from the corresponding author on reasonable request.

## Abbreviations

BMI: Body mass index
CVOT: Cardiovascular outcome trial
DPP4i: Dipeptidyl peptidase-4 inhibitors
GLP-1RA: Glucagon-like peptide-1 receptor agonists
SGLT2i: Sodium-glucose co-transporter-2 inhibitor
