# Supplementary material for: Generalizability of sodium-glucose co-transporter-2 inhibitors cardiovascular outcome trials to the type 2 diabetes population: a systematic review and meta-analysis
Source: Cardiovasc Diabetol. 2020 Jun 13;19:87. doi: 10.1186/s12933-020-01067-8 (PMC7293778; doi:10.1186/s12933-020-01067-8)
Supplement: Supplementary file 3 — Additional file 3. Enrollment criteria for sodium-glucose co-transporter-2 inhibitors cardiovascular outcome trials. [file 12933_2020_1067_MOESM3_ESM.docx]

**Additional file 3. Enrollment criteria for sodium-glucose co-transporter-2 inhibitors cardiovascular outcome trials.**

|  | CANVAS [14] | DECLARE-TIMI 58 [15] | EMPA-REG OUTCOME [16] | VERTIS CV [13] |
| --- | --- | --- | --- | --- |
| Type of diabetes | Type 2 | Type 2 | Type 2 | Type 2 |
| Age | ≥30 years with established cardiovascular disease  ≥50 years with two or more risk factors | ≥40 years with established cardiovascular disease  ≥55 years for males and ≥60 years with one or more risk factors | ≥18 years | ≥40 years |
| Body mass index | - | - | ≤45 kg/m^2^ | ≥18 kg/m^2^ |
| eGFR or ClCr | eGFR≥30 ml/min/1.73 m^2^ | ClCr≥60 ml/min/1.73 m^2^ | eGFR≥30 ml/min/1.73 m^2^ | eGFR≥30 ml/min/1.73 m^2^ |
| HbA1c | 7.0-10.5% | 6.5-12.0% | 7.0-10.0% | 7.0-10.5% |
| Cardiovascular disease | Established  cardiovascular disease or two risk factors (+ age) | Established  cardiovascular disease or one risk factor (+ age) | Established  cardiovascular disease | Established  cardiovascular disease |
|  |  |  |  |  |
| **Definition of established cardiovascular disease** |  |  |  |  |
| Stroke | ○ | ○ | ○ | ○ |
| Myocardial infarction | ○ | ○ | ○ | ○ |
| Percutaneous coronary intervention with or without stenting | ○ | ○ | ○ | ○ |
| Coronary artery bypass graft | ○ | ○ | ○ | ○ |
| Evidence of multi-vessel coronary artery disease |  | ○ | ○ |  |
| Evidence of single-vessel coronary artery disease |  |  | ○ |  |
| Unstable angina | ○ |  | ○ |  |
| Peripheral revascularization (angioplasty or surgery) | ○ | ○ | ○ | ○ |
| Symptomatic with documented hemodynamically-significant carotid or peripheral vascular disease | ○ | ○ | ○ | ○ |
| Amputation secondary to vascular disease | ○ | ○ | ○ | ○ |
|  |  |  |  |  |
| **Risk factors** |  |  |  |  |
| Duration of type 2 diabetes of 10 years or more | ○ |  | NA | NA |
| Hypertension | ○ | ○ | NA | NA |
| Cigarette smoker | ○ | ○ | NA | NA |
| Documented microalbuminuria or macroalbuminuria | ○ |  | NA | NA |
| Dyslipidemia according to low-density lipoprotein cholesterol |  | ○ | NA | NA |
| Dyslipidemia according to high-density lipoprotein | ○ |  | NA | NA |
