# Supplementary material for: Generalizability of sodium-glucose co-transporter-2 inhibitors cardiovascular outcome trials to the type 2 diabetes population: a systematic review and meta-analysis
Source: Cardiovasc Diabetol. 2020 Jun 13;19:87. doi: 10.1186/s12933-020-01067-8 (PMC7293778; doi:10.1186/s12933-020-01067-8)
Supplement: Supplementary file 4 — Additional file 4. Enrollment criteria evaluated in each study. [file 12933_2020_1067_MOESM4_ESM.docx]

**Additional file 4. Enrollment criteria evaluated in each study.**

|  | Birkeland, 2018 [19] | Canivell, 2019 [20] | Nicolucci, 2019 [21] | Shao, 2019 [22] | Wittbrodt, 2019 [23] |
| --- | --- | --- | --- | --- | --- |
| Age | Yes | Yes | Yes | Yes | Yes |
| Body mass index | Not assessed. | Yes | Yes | Yes | Yes |
| eGFR | Laboratory data were included only in a sensitivity analysis. | Yes | Yes | Yes | Yes |
| HbA1c | Laboratory data were included only in a sensitivity analysis. | Yes | Yes | Yes | Yes |
|  |  |  |  |  |  |
| **Definition of established cardiovascular disease** |  |  |  |  |  |
| Stroke | Yes | Yes | Yes | Yes | Yes |
| Myocardial infarction | Yes | Yes | Yes | Yes | Yes |
| Percutaneous coronary intervention with or without stenting | Yes | Yes | Yes | Yes | Yes |
| Coronary artery bypass graft | Yes | Yes | Yes | Yes | Yes |
| Evidence of multi-vessel coronary artery disease | Yes | Yes | Yes | Yes | Yes |
| Evidence of single-vessel coronary artery disease | Yes | Yes | Yes | Yes | Yes |
| Unstable angina | Yes | Yes | Yes | Yes | Yes |
| Peripheral revascularization (angioplasty or surgery) | Yes | Yes | Yes | Yes | Yes |
| Symptomatic with documented hemodynamically-significant carotid or peripheral vascular disease | Yes | Not reported | Yes | Yes | Yes |
| Amputation secondary to vascular disease | Yes | Not reported | Yes | Yes | Yes |
|  |  |  |  |  |  |
| **Risk factors** |  |  |  |  |  |
| Duration of type 2 diabetes of 10 years or more | Yes | Yes | Yes | Yes | Yes |
| Hypertension | Yes | Yes | Yes | Yes | Yes |
| Cigarette smoker | Not assessed. | Yes | Yes | Yes | Yes |
| Documented microalbuminuria or macroalbuminuria | Yes | Yes | Yes | Not reported | Yes |
| Dyslipidemia according to low-density lipoprotein cholesterol | Yes | Yes | Yes | Yes | Yes |
| Dyslipidemia according to high-density lipoprotein | Yes | Yes | Yes | Yes | Yes |
