# Supplementary material for: Generalizability of sodium-glucose co-transporter-2 inhibitors cardiovascular outcome trials to the type 2 diabetes population: a systematic review and meta-analysis
Source: Cardiovasc Diabetol. 2020 Jun 13;19:87. doi: 10.1186/s12933-020-01067-8 (PMC7293778; doi:10.1186/s12933-020-01067-8)
Supplement: Supplementary file 5 — Additional file 5. Publication bias. [file 12933_2020_1067_MOESM5_ESM.docx]

**Additional file 5. Publication bias.**

| Endpoint | Egger’s test |
| --- | --- |
| Head-to-head comparison of eligibility for the enrollment criteria of CANVAS versus DECLARE-TIMI 58 | 0.391 |
| Head-to-head comparison of eligibility for the enrollment criteria of CANVAS versus EMPA-REG OUTCOME | 0.477 |
| Head-to-head comparison of eligibility for the enrollment criteria of CANVAS versus VERTIS-CV | 0.972 |
| Head-to-head comparison of eligibility for the enrollment criteria of DECLARE-TIMI 58 versus EMPA-REG OUTCOME | 0.853 |
| Head-to-head comparison of eligibility for the enrollment criteria of DECLARE-TIMI 58 versus VERTIS-CV | 0.599 |
| Head-to-head comparison of eligibility for the enrollment criteria of EMPA-REG OUTCOME versus VERTIS-CV | 0.431 |
