# Supplementary material for: Generalizability of sodium-glucose co-transporter-2 inhibitors cardiovascular outcome trials to the type 2 diabetes population: a systematic review and meta-analysis
Source: Cardiovasc Diabetol. 2020 Jun 13;19:87. doi: 10.1186/s12933-020-01067-8 (PMC7293778; doi:10.1186/s12933-020-01067-8)
Supplement: Supplementary file 6 — Additional file 6. Sensitivity analysis. [file 12933_2020_1067_MOESM6_ESM.docx]

**Additional file 6. Sensitivity analysis.**

| Endpoint | Birkeland, 2018 [19] | Canivell, 2019 [20] | Nicolucci, 2019 [21] | Shao, 2019 [22] | Wittbrodt, 2019 [23] |
| --- | --- | --- | --- | --- | --- |
| Head-to-head comparison of eligibility for the enrollment criteria of DECLARE-TIMI 58 versus CANVAS(OR) | **1.53** | **1.82** | 1.51 | **2.14** | **1.77** |
| Head-to-head comparison of eligibility for the enrollment criteria of CANVAS versus EMPA-REG OUTCOME (OR) | **3.30** | **2.64** | **2.92** | **2.51** | **3.60** |
| Head-to-head comparison of eligibility for the enrollment criteria of CANVAS versus VERTIS-CV (OR) | **2.69** | - | **2.65** | **2.06** | **3.34** |
| Head-to-head comparison of eligibility for the enrollment criteria of DECLARE-TIMI 58 versus EMPA-REG OUTCOME (OR) | **5.04** | **4.79** | **4.41** | **5.35** | **6.35** |
| Head-to-head comparison of eligibility for the enrollment criteria of DECLARE-TIMI 58 versus VERTIS-CV (OR) | **4.18** | - | **4.06** | **5.01** | **6.38** |
| Head-to-head comparison of eligibility for the enrollment criteria of EMPA-REG OUTCOME versus VERTIS-CV (OR) | 0.92 | - | 1.07 | 1.02 | 1.02 |

Values with p<0.05 are in bold text.
