# Supplementary material for: Generalizability of sodium-glucose co-transporter-2 inhibitors cardiovascular outcome trials to the type 2 diabetes population: a systematic review and meta-analysis
Source: Cardiovasc Diabetol. 2020 Jun 13;19:87. doi: 10.1186/s12933-020-01067-8 (PMC7293778; doi:10.1186/s12933-020-01067-8)
Supplement: Supplementary file 7 — Additional file 7. Estimated representativeness of the analytical cohorts of included studies. [file 12933_2020_1067_MOESM7_ESM.docx]

**Additional file 7. Estimated representativeness of the analytical cohorts of included studies.**

| First Author, year | Included subjects (*10^3^) | Country | Population (*10^3^) ^a^ | Prevalence of type 2 diabetes (%) ^b^ | Patients with type 2 diabetes (*10^3^) ^c^ | Estimated representativeness (%) ^d^ |
| --- | --- | --- | --- | --- | --- | --- |
| Birkeland, 2018 [19] | 804 | Germany | 82 906 | 7.4 | 6 135 | 9.7 |
|  |  | the Netherlands | 17 232 | 6.1 | 1 051 |  |
|  |  | Norway | 5 312 | 6.6 | 351 |  |
|  |  | Sweden | 10 175 | 6.9 | 702 |  |
| Canivell, 2019 [20] | 373 | Spain | 46 796 | 9.4 | 4 399 | 8.5 |
| Nicolucci, 2019 [21] | 342 | Italy | 60 422 | 8.5 | 5 136 | 6.6 |
| Shao, 2019 [22] | 12 | Taiwan | NA | NA | NA | NA |
| Wittbrodt, 2019 [23] | 173 | the United States of America | 326 687 | 9.1 | 29 729 | 0.6 |

Legend: ^a^ According to the World Health Organization (<https://data.worldbank.org/indicator/sp.pop.totl>); ^b^ According to the World Health Organization (<https://www.who.int/diabetes/country-profiles/en/>); ^c^ Estimated as Population x Prevalence of type 2 diabetes; ^d^ Estimated as Included subjects divided by Patients with type 2 diabetes. Databases were accessed on May 02, 2020
